# Frog Skin Peptides Hylin-a1, AR-23, and RV-23: Promising Tools Against Carbapenem-Resistant *Escherichia coli* and *Klebsiella pneumoniae* Infections

**DOI:** 10.3390/antibiotics14040374

**Published:** 2025-04-03

**Authors:** Annalisa Chianese, Annalisa Ambrosino, Rosa Giugliano, Francesca Palma, Preetu Parimal, Marina Acunzo, Alessandra Monti, Nunzianna Doti, Carla Zannella, Massimiliano Galdiero, Anna De Filippis

**Affiliations:** 1 Department of Experimental Medicine, Università Degli Studi Della Campania Luigi Vanvitelli, 80138 Naples, Italy; annalisa.chianese@unicampania.it (A.C.); annalisa.ambrosino@unicampania.it (A.A.); rosa.giugliano@unicampania.it (R.G.); francesca.palma@unicampania.it (F.P.); preetu.parimal@unicampania.it (P.P.); marina.acunzo@unicampania.it (M.A.); carla.zannella@unicampania.it (C.Z.); massimiliano.galdiero@unicampania.it (M.G.); 2 Institute of Biostructures and Bioimaging (IBB), National Research Council (CNR), 80134 Naples, Italy; alessandra.monti@ibb.cnr.it (A.M.); nunzianna.doti@cnr.it (N.D.); 3 Complex Operative Unit of Virology and Microbiology, University Hospital of Campania “Luigi Vanvitelli”, 80138 Naples, Italy

**Keywords:** antimicrobial peptides, carbapenem-resistant strains, *Escherichia coli*, *Klebsiella pneumoniae*, peptides, antibacterial, multi-drug resistance

## Abstract

Background/Objectives. One of the pressing challenges in global public health is the rise in infections caused by carbapenem-resistant *Enterobacteriaceae.* Growing bacterial drug resistance, coupled with the slow development of new antibiotics, highlights the critical need to explore and develop new broad-spectrum antimicrobial agents able to inhibit bacterial growth efficiently. In recent years, antimicrobial peptides (AMPs) have gained significant attention as a promising alternative to conventional drugs, owing to their antimicrobial potency, low toxicity, and reduced propensity for fostering resistance. Our research aims to investigate the antibacterial ability of three amphibian AMPs, namely Hylin-a1, AR-23, and RV-23, against both antibiotic-sensitive and carbapenem-resistant strains of *Escherichia coli* and *Klebsiella pneumoniae*. Methods. A 3-(4,5 dimethylthiazol-2-yl)-2,5-diphenyltetrazolium bromide assay (MTT) was performed to identify non-cytotoxic concentrations of peptides. A microdilution assay evaluated the antibacterial effect, determining the peptides’ minimum inhibitory concentration (MIC). In addition, the checkerboard test analyzed the compounds’ synergistic effect with meropenem. Results. We demonstrated that peptides with low toxicity profile and resistance to proteolytic activity exhibited strong antibacterial activity, with MIC ranging from 6.25 to 25 μM. The antibiofilm mechanism of action of peptides was also investigated, suggesting that they had a crucial role during the biofilm formation step by inhibiting it. Finally, we highlighted the synergistic effects of peptides with meropenem. Conclusions. Our study identifies Hylin-a1, AR-23, and RV-23 as promising candidates against Gram-negative bacterial infections with a favorable therapeutic profile. This effect could be related to their great flexibility, as evidenced by circular dichroism data, confirming that the peptides could assume an α-helical conformation interacting with bacterial membranes.

## 1. Introduction

The continued rise in bacterial infections and misuse of antibacterial drugs threaten global public health. Hospital admissions, high medical costs, and bacterial infection mortality are increasingly taking over. According to the World Health Organization (WHO), about 4.95 million deaths are reported every year worldwide related to antimicrobial resistance (AMR), with 133,000 people dying only in Europe from direct causes and 541,000 for indirect causes [1]. Since its initial publication in 2017 and subsequent updates over the past year, the World Health Organization has established a Bacterial Priority Pathogens List (BPPL). This classification organizes bacteria into three priority levels—critical, high, and medium—according to their antibiotic resistance profiles. [2]. To date, 24 pathogens from 15 different bacterial families have been included [3]. Among these, Gram-negative bacteria exhibit the most extensive range of antibiotic resistance. The most worrying pathogens are carbapenem-resistant *Enterobacterales* (CRE), which produce carbapenemase, an enzyme that hydrolyzes β-lactam antibiotics, leading to resistance [4,5]. Until 2001, most clinically isolated enterobacteria had a high carbapenem sensitivity profile [6,7]. However, since 2015, the resistance rate has increased drastically by more than 60% [7]. As widely documented, there is a high global frequency of *Klebsiella pneumoniae* (*K. pneumoniae*) and *Escherichia coli* (*E. coli*) resistant to carbapenem indicated as CRKP and CREco, respectively [8,9,10,11,12,13]. In particular, carbapenemases can be divided into different classes depending on the geographical distribution: classes B (VIM) and D (OXA-48) are common in Europe [14,15,16,17,18], class A carbapenemases are predominantly found in the Americas, whereas class B enzymes, such as NDM and IMP, are commonly distributed across India, China, and Australia [19]. Most hospital-acquired infections are estimated to be related to bacterial infections caused by *K. pneumoniae* and *E. coli* [20]. These bacteria mainly colonize the intestinal tract, but are sometimes responsible for urinary tract infections, bloodstream infections, and lung infections, particularly in patients with compromised immune systems [21,22]. These infections can be life-threatening and complicate treatment, often resulting in extended hospital stays.

The rapid spread of such a large variety of resistant bacteria and, on the other hand, the lack of novel antibiotics, require the search for alternative antimicrobial agents derived from natural sources. For instance, antimicrobial peptides (AMPs) are a group of peptides isolated from plants, algae, animals, and microorganisms. Among animals, frogs are the richest source of AMPs, producing over 1000 different types in their skin secretions to adapt and thrive in their challenging habitats [23,24,25,26,27]. These peptides share several physio-chemical characteristics: small length (ranging from 10 to 50 amino acid residues), hydrophobic nature, cationic charge, and amphipathic alpha-helical structure [26]. All these features are essential for their well-documented bioactivity [26]. They can interact with membranes, including the bacterial surface, crossing it and inducing cell lysis [28,29,30,31,32]. Well-documented examples are the temporins, peptides secreted by *Rana temporaria*. They are particularly active against Gram-positive bacteria, showing a minimum inhibitory concentration (MIC) ranging from 2.5 to 20 μM [33]. Our research team has documented the broad-spectrum efficacy of [Pro3, DLeu9] temporin L derivatives against Gram-negative bacteria [34] and some human viruses [26], showing the putative effect of these peptides against bacterial lipopolysaccharide (LPS)’s outer membrane and viral envelope. Other studies report the antimicrobial efficacy of different frog-derived peptides. For instance, HS-1 has demonstrated significant activity against Gram-positive bacteria at concentrations ranging from 11 to 46 μM [35]. Additionally, esculentin exhibits a broad spectrum of activity against multidrug-resistant strains, including *Staphylococcus aureus*, *Acinetobacter baumannii*, *Stenotrophomonas maltophilia*, and *Pseudomonas aeruginosa* [36,37,38,39].

Our group also discovered an amphibian peptide named Hylin-a1, which shows a broad-spectrum activity against pathogens. It interferes with the membrane of *S. aureus*, including clinical isolates [40], and the envelopes of various viruses [41,42,43]. Considering the encouraging results achieved so far with the use of amphibian AMPs in treating bacterial infections, this study aims to assess the inhibitory effects of three lesser-studied peptides—Hylin-a1, AR-23, and RV-23—which are derived from the skin of *Rana tagoi*, *Rana draytonii*, and *Hypsiobas albopunctatus* (*H. albopunctatus*), respectively, against Gram-negative bacteria. The peptides demonstrate significant inhibitory activity against *E. coli* and *K. pneumoniae* growth. Few studies have evaluated the antibacterial activity of the three selected peptides against Gram-negative bacteria. In detail, Zang et al. reported the inhibitory effect of melittin-related peptides (AR-23 and RV-23) against *E. coli* and *K. pneumoniae* [44]. Additionally, a separate study reported that Hylin-a1 effectively inhibited the growth of *E. coli*, although its activity against *K. pneumoniae* was not evaluated [45]. However, the primary focus of this study is to evaluate and emphasize the inhibitory effects of the selected AMPs against the carbapenem-resistant clinical isolates of enterobacteria. Our results underscore the strong antibacterial activity of these peptides and offer insights into their potential mechanism of action. Specifically, their antibacterial effects appear to be mediated through interactions with the bacterial membrane, as evidenced by structural studies using circular dichroism to analyze the peptides in complexes with LPS. Overall, these findings suggest that Hylin-a1, AR-23, and RV-23 could be promising candidates as AMPs, potentially contributing to the fight against the growing issue of AMR.

## 2. Results

### 2.1. Cytotoxic Activity

**Figure 1:**
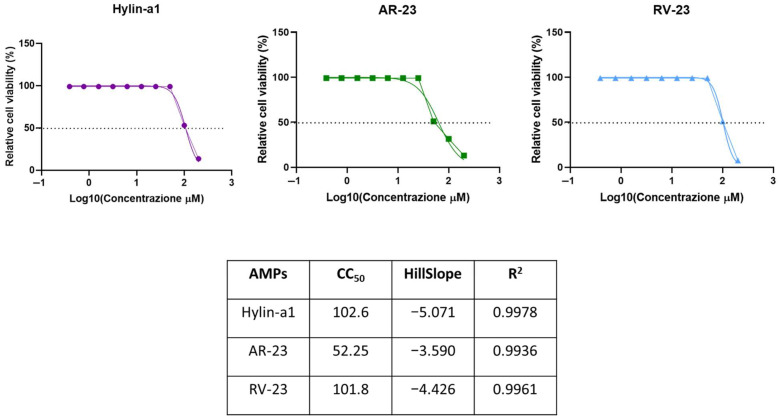
Dose–response curves for the AMPs against HaCaT cell line and CC_50_ (reported in μM), HillSlope, and R^2^ values were obtained by the fitting of the curves. The viability effect of Hylin-a1, AR-23, and RV-23 was analyzed on HaCaT cells. The cell monolayer was treated with serial dilutions of peptides for 20 h. Untreated cells were used as a positive control, while dimethyl sulfoxide (DMSO) was used as a negative control. HillSlope: The measure of the steepness of the dose–response curve. A positive value indicates a cooperative response, while a negative value suggests an inhibitory effect. R^2^ (coefficient of determination) indicates how well the model fits the data. Ranges from 0 to 1, with 1 representing a perfect fit. Higher R^2^ values suggest a more accurate prediction of the biological response to varying concentrations [46].

Hylin-a1, AR-23, and RV-23 were synthesized utilizing the Fmoc (fluorenylmethyloxycarbonyl) solid-phase peptide synthesis strategy and purified to homogeneity (see the Section 4 for details). To evaluate their safety profile, cytotoxicity assays were performed on HaCaT keratinocytes. Cells were exposed for 20 h to peptides at doses ranging from 0.39 to 100 μM. The data presented in Figure 1 show that Hylin-a1 and RV-23 have lower toxicity with a 50% cytotoxic concentration (CC_50_) of 100 μM. In contrast, AR-23 exhibits a CC_50_ of 50 μM.

### 2.2. Antibacterial Activity of Hylin-a1, AR-23, and RV-23

The three peptides were tested for their antibacterial activity. Accordingly to their safety profiles (Figure 1), the potential antibacterial activity of peptides, in terms of MIC and MBC values, was evaluated against Gram-negative bacterial strains, such as *K. pneumoniae* ATCC 10031 and *E. coli* ATCC 25992, in the concentration range from 50 to 1.56 μM. As described in the Materials and Methods section, the antibacterial effect of peptides was investigated using a microdilution assay. Each concentration of AMPs was inoculated with bacteria in 96-well plates and incubated at 37 °C for 20 h. After that, MIC values were determined by analyzing absorbance and expressed as a percentage of inhibition. MBC was assessed by observing the colonies’ growth on agar plates, as described elsewhere [40]. The values reported in Table 1 demonstrate that all three peptides interfere with bacterial growth with MIC values equal to 6.25 μM against *K. pneumoniae* ATCC 10031, and 12.5 μM for RV-23 and 25 μM for the other two against *E. coli* ATCC 25992. However, the MBC values determined are very different from each other, both between the three peptides towards the same bacterium and between the two bacteria.

In summary, the peptides demonstrate greater efficacy against *K. pneumoniae* than *E. coli*, with RV-23 showing the best activity profile against both bacterial strains. The data presented in Table 2 indicate that all of the selected peptides possess antibacterial properties against numerous resistant strains.

This analysis was further expanded to include a broad range of clinical isolates, characterized by different carbapenem resistance profiles and sources of isolation (refer to Table 3). 

Once again, RV-23 is the most active, with the best antibacterial activity against both *K. pneumoniae* clinical isolates and *E. coli* with MIC and MBC values between 6.25 and 50 μM. Instead, AR-23 and Hylin-a1 show reduced inhibitory activity in blocking bacterial growth, and bactericidal activity is drastically reduced or not even highlighted in our experimental conditions. In detail, RV-23 showed a bactericidal effect against all clinical isolates, except for *K. pneumoniae* 1746 and *E. coli* 70028. AR-23 is bactericidal only against *E. coli* 716 and *E. coli* 1376 strains, with MBC at 50 μM. Finally, Hylin-a1 shows bactericidal activity only against *K. pneumoniae* 1711, *K. pneumoniae* 1745, and *E. coli* 11228 with MBC at 50 μM.

### 2.3. Time–Kill Assay

**Figure 2:**
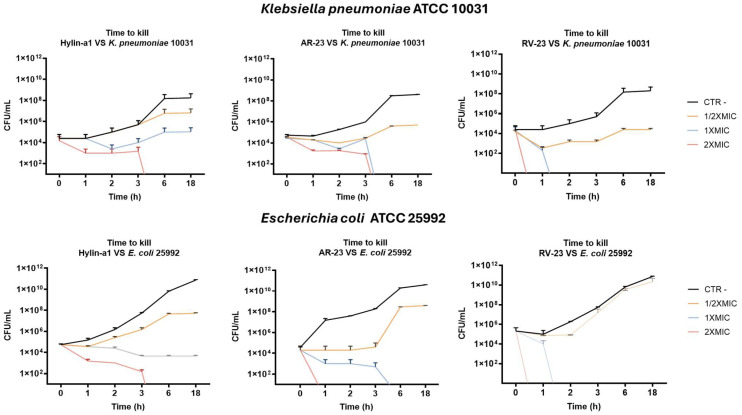
Evaluation of the kinetic-killing effect of Hylin-a1, AR-23, and RV-23 against *K. pneumoniae* and *E. coli.* 2× MIC, 1× MIC, and 1/2 MIC were tested for each peptide in a range of time from 0 to 18 h. Untreated bacteria was used as control negative (CTR- in black).

The bactericidal activity of Hylin-a1, AR-23, and RV-23 was further evaluated using the time–kill assay against *K. pneumoniae* ATCC 10031 and *E. coli* ATCC 25992. This in vitro method allows for assessing the kinetics of bacterial killing by peptides over time. Bacterial cultures were treated with different peptide concentrations (2× MIC, 1× MIC, and 1/2× MIC), and bacterial viability was monitored at defined time intervals. Specifically, at each time point, 50 μL aliquots of the bacterial–peptide mixtures were collected and plated on agar (see Materials and Methods for further details). Following incubation, colony-forming units (CFUs) were counted and the killing rate of each peptide concentration was calculated (Figure 2).

In line with previous results, RV-23 is the most active antibacterial peptide exhibiting a significant reduction in bacterial viability already in the first 2 h at 2× MIC (12.5 μM for *K. pneumoniae* and 25 μM for *E. coli*) and 1× MIC (6.25 μM for *K. pneumoniae* and 12.5 μM for *E. coli*). AR-23 shows a bactericidal effect at 2× MIC (12.5 μM for *K. pneumoniae* and 25 μM for *E. coli*) and 1× MIC (6.25 μM for *K. pneumoniae* and 25 μM for *E. coli*) after 3 h. At the same time, also Hylin-a1 kills bacteria, albeit at 2× MIC (12.5 and 50 μM for *K. pneumoniae* and *E. coli*, respectively).

### 2.4. Antibiofilm Activity

**Figure 3:**
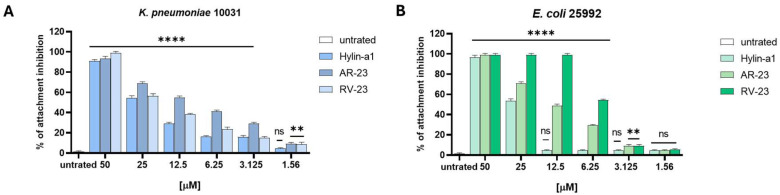
The effect of Hylin-a1, AR-23, and RV-23 on the initial cell attachment of biofilm formation. (**A**) *K. pneumoniae ATCC* 10031, (**B**) *E. coli ATCC* 25992. The bacterial suspension was exposed on the surface together with peptides (50–1.56 μM), and, after 2 h, the non-adherent cells were removed while adherent cells were quantified by CV staining by reading absorbance at 570 nm. Statistical analysis was performed by One-way ANOVA followed by Dunnett’s multiple comparisons test. Significances are referred to the untreated sample. **** *p* < 0.0001; ** *p* = 0.0059; ns: non-significant.

We also investigated the ability of peptides to interfere with biofilm using three different assays (refer to the Section 4) against *K. pneumonia* and *E. coli*. We observed that none of the peptides exhibited inhibitory effects on biofilm formation or degradation (Supplementary Materials, Figure S1). On the other hand, they showed a strong anti-biofilm activity in the initial stage of cell attachment. As reported in Figure 3, RV-23 causes a total reduction in biofilm attachment at 50 and 12.5 μM for *K. pneumoniae* and *E. coli*, respectively. Conversely, AR-23 and Hylin-a1 reduce biofilm at 50 μM by 80–100% for both bacteria.

### 2.5. Synergistic Effect of Hylin-a1, AR-23, and RV-23 with Meropenem

**Figure 4:**
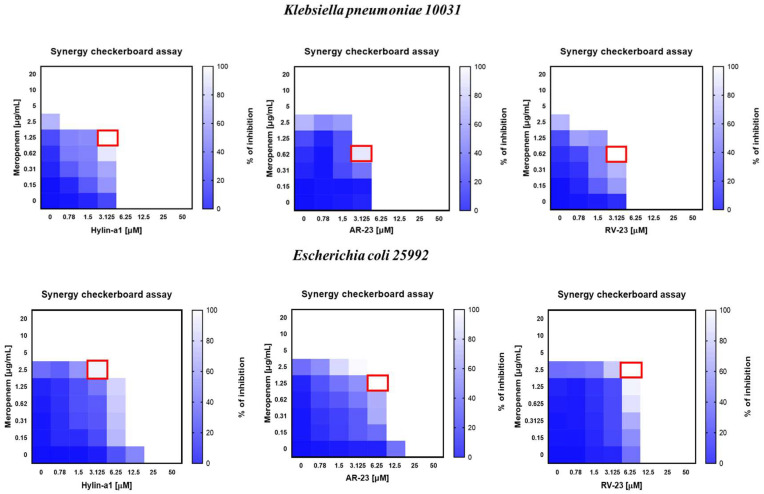
Evaluation of the synergism between peptides and meropenem using a checkerboard assay [47]. The rows contain two-fold dilutions of meropenem, while we reported a two-fold serial dilution of AMPs in the columns. The data are expressed as a percentage of inhibition of bacteria growth, while the red square indicates synergism with the lowest FICI.

The synergistic effect of the selected peptides and meropenem was examined using a checkerboard assay. This method investigates the interaction between various antimicrobial agents and their combined effectiveness against bacteria. The checkerboard test is widely used in antimicrobial research [47]. It determines whether the combined agents act synergistically, additively, or antagonistically to identify combinations that can reduce drug resistance. The data in Figure 4 evidence that all of the combinations tested against *K. pneumoniae* and *E. coli* determine a partial synergism between each peptide and the drug, with FICI (Fractional Inhibitory Concentration Index) values ranging from 0.5 to 1 μM.

As reported, the MIC of meropenem is 5 μg/mL against both bacteria. When the antibiotic was combined with each peptide, we observed a strong decrease in the MIC value. According to the best antimicrobial activity evidenced for RV-23, its combination with meropenem provides the best results, with a MIC at 3.125 μM (peptide) and 0.625 μg/mL (antibiotic) against *K. pneumoniae*, and 6.25 μM (peptide) and 2.5 μg/mL (antibiotic) against *E. coli*. Surprisingly, AR23, which works worse than RV-23 when used alone, when combined with the antibiotic, gives similar if not better results than RV-23, indeed, with calculated MIC values at 3.125 μM (peptide) and 0.625 μg/mL (antibiotic) against *K. pneumoniae*, and 6.25 μM (peptide) and 1.25 μg/mL (antibiotic) against *E. coli*. Regarding the combination of Hylin-a1 and meropenem, we observed significant activity only against *E. coli*, with MIC at 3.125 μM (peptide) and 2.5 μg/mL (antibiotic).

### 2.6. CD Analysis of Hylin-a1, AR-23, and RV-23 with LPS

**Figure 5:**
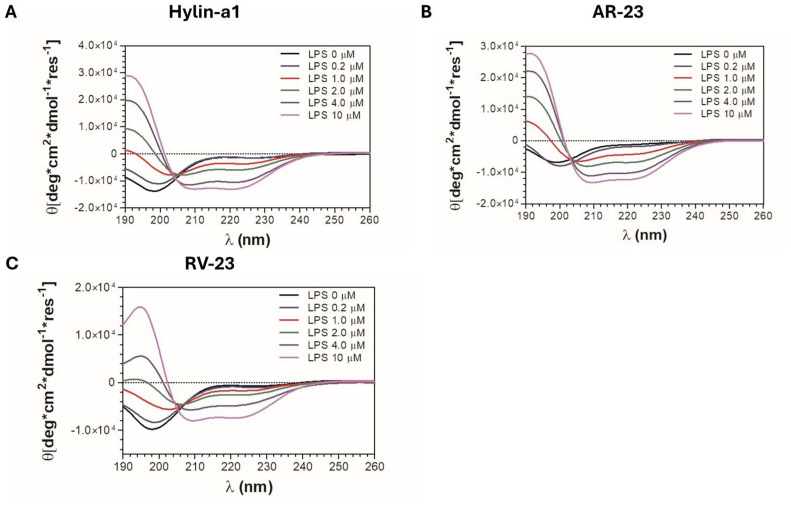
The overlay of representative CD spectra for Hylin-a1 (**A**), AR-23 (**B**), and RV-23 (**C**) is presented, showing their profiles at a concentration of 50 μM and in the presence of an increasing amount of LPS, ranging from 0.2 to 10 μM, in 5 mM sodium phosphate buffer at pH 7.4. CD spectra were recorded from 190 to 260 nm using a JASCO J-705 CD spectrophotometer at a controlled temperature of 25 °C (for further details, see Section 4). The spectra were adjusted by subtracting the respective background signals. Graphical representations were generated using GraphPad Prism 5.1 software (GraphPad Software, San Diego, CA, USA).

The conformational behavior of peptides in solution were assessed using circular dichroism (CD) spectroscopy, as described in the Section 4 (Materials and Methods). As illustrated in Figure 5A–C (black lines), CD spectra are characterized by a prominent negative minimum near 198 nm and an additional negative peak at 190 nm, demonstrating that the peptides exhibit remarkable flexibility in solution. CD spectroscopy was also employed to investigate the impact of LPS on the secondary structures of the peptides. Peptides were titrated with increasing amount of *E. coli* O128:B12 LPS, and the resulting changes in the secondary structure of the peptides were monitored. Notably, the addition of LPS resulted in two distinct CD bands at approximately 208 nm and 222 nm, accompanied by a positive band at 190 nm (Figure 5).

These observations indicate a transition of the peptides from a disordered state toward an α-helical conformation in the presence of LPS. Moreover, increasing concentrations of LPS resulted in CD spectra exhibiting progressively deeper absolute minima, indicative of a dose-dependent enhancement in the structural organization of peptides. In conclusion, the results offer preliminary insights into the ability of the peptides to adopt an alpha-helical conformation in the presence of lipopolysaccharides, as this secondary structure is well known to be closely linked to their ability to interact with bacterial membranes and their antimicrobial activity [26].

### 2.7. Serum Stability of Hylin-a1, AR-23, and RV-23

The susceptibility of Hylin-a1, AR-23, and RV-23 to proteolytic degradation in serum was evaluated, following the protocol reported in Section 4.3.

**Figure 6:**
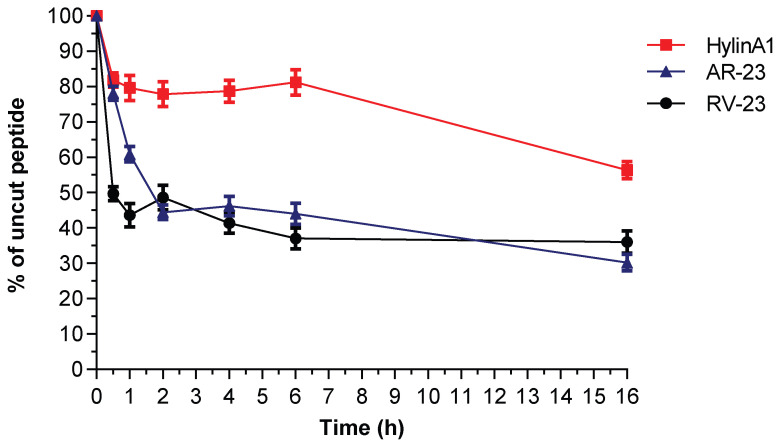
AMP stability in serum. Stability profiles of Hylin-a1 (red line), AR-23 (gray line), and RV-23 (black line) are reported. All stability tests were performed at least in triplicate. LC-MS analyses were performed using an Agilent 1260 Infinity II LC/MSD system (Milan, Italy). The percentages of intact peptides after different time points of incubation in serum were determined by integrating RP-HPLC peaks from the chromatograms recorded at a wavelength of 210 nm. The test was performed in triplicate, and the data are presented as mean values, with error bars representing standard deviations.

Under our experimental conditions, AR-23 and RV-23 are rapidly degraded, showing half-lives of less than 2 h (Figure 6, blue and black lines). However, over the following hours, the percentage of intact fragments remains almost constant (around 30%) for both peptides up to 24 h. In contrast, Hylin-a1 shows resistance to proteolytic degradation. While a slight decrease in the content of the intact peptide in solution is observed after 2 h, the decrease does not continue over the following 7 h (Figure 6, red line). Note that, after 16 h, 60% of the intact peptide remains in the solution and 50% at 24 h, underlining the significant resistance of Hylin-a1 to degradation by proteases. Based on these data, subsequent studies were conducted for up to 24 h.

## 3. Discussion

Bacterial resistance to commonly used antibiotics has become a major global challenge in the 21st century, underscoring the urgent need for new and effective agents to combat drug resistance. In this context, AMPs have emerged as a promising alternative to conventional antibiotics [48]. Naturally derived AMPs possess several advantageous properties: they are relatively easy and cost-effective to synthesize due to their natural origin and widespread availability, unlike many synthetic antibiotics. Furthermore, AMPs exhibit a lower propensity for inducing resistance and demonstrate a remarkable ability to penetrate bacterial membranes, exerting their antimicrobial effects through multiple mechanisms [49]. Several studies have documented that frogs are a rich source of AMPs. This abundance is largely attributed to their constant exposure to pathogens, predators, and other environmental stressors, which has driven the evolution of a diverse array of bioactive compounds produced by specialized glands through a holocrine secretion mechanism. Among the most studied amphibian-derived AMPs are dermaseptins [49,50,51,52], esculentins [37,53,54], magainins [55,56], temporins [26,34,57,58,59], and brevinins, all recognized for their broad-spectrum biological activities and potential as leads for new drug development. In this study, we specifically focused on three amphibian peptides: (i) Hylin-a1, first isolated in 2009 from *H. albopunctatus*, known for its inhibitory activity against Gram-positive bacteria [40,45], and (ii) AR-23 and (iii) RV-23, both members of the *Ranidae* family, which have been extensively investigated for their ability to inhibit a variety of human and animal pathogenic viruses [43].

These three peptides have been a longstanding focus of investigation within our research group. In this study, we expanded our analysis to provide a comparative evaluation of the antimicrobial activities of Hylin-a1, AR-23, and RV-23 against a variety of Gram-negative bacterial strains, including MDR clinical isolates and biofilm-producing bacteria. Given the limited existing research on the activity of these peptides against Gram-negative pathogens, our findings contribute valuable new insights into this area. Our results demonstrate that Hylin-a1, AR-23, and RV-23 exhibit significant inhibitory effects against standard reference strains, with MIC values in the low micromolar range. Their antimicrobial action is observed within a few hours at concentrations of 1× MIC and 2× MIC. Among the three peptides, RV-23 displayed the most potent antimicrobial profile against both *E. coli* and *K. pneumoniae*. Notably, we also show for the first time that these peptides have antibacterial activity against clinically isolated MDR strains (see Table 2). In particular, RV-23 stands out with a therapeutic index (TI) of 16 against *K. pneumoniae* and 8 against *E. coli*, coupled with a faster action. While Hylin-a1 and AR-23 show similar antibacterial activity, AR-23 is distinguished by its quicker bactericidal effect. Additionally, the three selected peptides exhibited antibiofilm activity (Figure 3) by interfering with the initial stage of bacterial cell attachment. AR-23 and Hylin-a1 showed MICs of 50 μM against both bacterial strains, while RV-23 displayed MICs of 50 μM and 12.5 μM against *K. pneumoniae* and *E. coli*, respectively. Our biological findings are supported by circular dichroism (CD) analyses, which revealed conformational changes in the peptide structures upon interaction with LPS, suggesting a direct action on bacterial surfaces (Figure 5). Moreover, the CD data provide preliminary evidence that these peptides can adopt an alpha-helical conformation, a secondary structure commonly associated with antimicrobial activity. It is well-established that the alpha-helical structure of AMPs plays a critical role in their function [60,61,62]. This structure is often amphipathic, which is essential for the peptides’ ability to interact with and insert into the lipid bilayer of microbial membranes. This mechanism is particularly effective against bacteria with more fluid and less rigid membranes, such as Gram-negative bacteria. Once inserted into the membrane, the peptides may aggregate to form larger structures, such as pores, leading to leakage of cellular contents. Alternatively, the peptides may cause membrane thinning, curvature, or even complete rupture, ultimately resulting in the death of the microorganism.

The activity of AR-23 and RV-23 against Gram-negative bacteria has been briefly described in previous studies. Zhang et al. [63] evaluated the antibacterial effects of both peptides against *E. coli*. They reported MIC values of 22.67 μM for AR-23 and 3.33 μM for RV-23 [63], which are consistent with our findings, confirming RV-23 as the most potent of the two. The authors attributed this enhanced activity to RV-23’s lower hydrophobicity and higher hydrophobic moment, factors strongly associated with reduced cytotoxicity and greater selectivity compared to AR-23. In contrast, the antibacterial properties of Hylin-a1 have been investigated more extensively, often through the design of peptide analogs. For example, Park et al. [64] recently reported the activity of Hylin-a1 analogs against *A. baumannii*, including carbapenem-resistant strains, with remarkably low MIC values (2–16 μM) [64]. Consistent with our observations, these studies also demonstrated that Hylin-a1, AR-23, and RV-23 exert their antibacterial effect by binding to LPS and disrupting the bacterial membrane.

Our second objective was to identify compounds capable of replacing or enhancing the effects of conventional antibiotics, particularly against Gram-negative bacteria that have developed multiple resistance mechanisms. We assessed the effect of each peptide in combination with meropenem against *K. pneumoniae* and *E. coli*, yielding promising results (Figure 4). Meropenem was selected due to its broad-spectrum activity, targeting both Gram-positive and Gram-negative bacteria by penetrating their membranes and binding to penicillin-binding proteins (PBPs), which play a crucial role in bacterial cell wall biosynthesis [65]. Among the combinations tested, RV-23 with meropenem proved to be the most effective, demonstrating a significantly enhanced inhibitory effect against both bacterial strains. The combination of AR-23 with meropenem also produced positive results, although to a lesser extent than RV-23. Lastly, the combination of Hylin-a1 and meropenem showed partial synergism against *E. coli*, but was less effective against *K. pneumoniae*.

## 4. Materials and Methods

### 4.1. Synthesis and Characterization of Peptides

Protected amino acids, coupling agents (HATU, Oxyma) used for peptide synthesis were purchased from Merck (Milan, Italy), and Fmoc-Rink Amide AM resin was purchased from Novabiochem (Milan, Italy). Synthesis reagents, including acetonitrile (CH_3_CN), dimethylformamide (DMF), N,N′-Diisopropylcarbodiimide (DIC), tri-isopropylsilane (TIS), trifluoroacetic acid (TFA), sym-collidine, di-ethyl-ether, diisopropylethylamine (DIPEA), and piperidine were purchased from Merck (Milan, Italy).

The C-terminal amidated peptides AR-23 (H-AIGSILGALAKGLPTLISWIKNR-NH2), RV-23 (H-RIGVLLARLPKLFSLFKLMGKKV-NH2), and Hylin-a1 (H-IFGAILPLALGALKNLIK-NH2) were synthesized on solid-phase using Rink-Amide AM resin with a substitution rate of about 0.60 mmol/g. The synthesis followed a standard Fmoc peptide protocol, employing Oxyma-DIC and HATU-collidine as coupling reagents, in accordance with previously established methodologies [43]. To cleave the peptides from the solid support, the resin was treated with a TFA/TIS/H_2_O mixture (95:2.5:2.5, *v*/*v*/*v*) for 3 h at room temperature. The crude peptides were subsequently precipitated using cold diethyl ether, dissolved in a H2O/CH_3_CN (75:25, *v*/*v*) solution, and then lyophilized. Purification was conducted using a WATERS 2545 preparative system (Waters, Milan, Italy), equipped with a WATERS 2489 UV/Visible detector. A linear gradient from 5% to 70% CH_3_CN containing 0.1% TFA in water was applied over 20 min at a flow rate of 12 mL/min, utilizing a Jupiter C18 column (5 μm, 300 Å, 150 × 21.2 mm ID). The absorbance was monitored at 214 nm. Mass spectrometry (MS) characterization of the synthesized peptides was performed using an Agilent 1290 Infinity LC System coupled with an Agilent 6230 time-of-flight (TOF) LC/MS System (Agilent Technologies, Cernusco sul Naviglio, Italy). The system included a photodiode array (PDA) detector, a binary solvent pump with a degasser, a column heater, and an autosampler. The LC-MS analysis utilized a C18 Waters xBridge column (5 μm, 2.1 × 50 mm) with a linear gradient of CH_3_CN/0.05% TFA in water (0.05% TFA) from 5% to 70% over 10 min at a flow rate of 0.2 mL/min.

The yields of the target peptides were calculated as the ratio of the experimental weight of the pure peptide to the theoretical weight based on the synthesis scale used, yielding an estimated yield of approximately 70%. The relative purity of the peptides was determined from the peak area of the target peptide relative to the sum of the areas of all detected peaks in the UV chromatograms at 210 nm, with purities reaching up to 95% [43,45].

### 4.2. Cell Culture and Viability Assay

The cytotoxic effect of peptides was examined using 3-(4,5 dimethylthiazol-2-yl)-2,5-diphenyltetrazolium bromide (MTT), as previously described [40]. Cell viability, expressed as a percentage, was calculated in relation to the viability of untreated cells (+). DMSO was used as negative control (−). The human keratinocyte cell line HaCaT (Cell Lines Service, CLS-, Eppelheim, Germany) was used as a cellular model to evaluate the peptides’ cytotoxicity. HaCaT cells were grown in Dulbecco’s Modified Eagle Medium (DMEM) with 4.5 g/L of glucose (Microtech, Naples, Italy) supplemented with an antibiotic solution 100× (Himedia, Naples, Italy) and 10% Fetal Bovine Serum (FBS, Microtech).

### 4.3. Antibacterial Activity

#### 4.3.1. Bacterial Strains

*K. pneumoniae* ATCC 10031 and *E. coli* ATCC 25992 were used as bacterial models to evaluate the antibacterial activity of AMPs. Each strain was grown in Mueller Hinton Broth (MH Broth), and 5 × 10^5^ CFU/mL was used to evaluate antibacterial activity. Clinical carbapenem-resistant *K. pneumoniae* and *E. coli* isolates were collected at the AOU of Virology and Microbiology of the University of Campania “Luigi Vanvitelli”. Their resistance profile and source of isolation are indicated in Table 3 and Table S1 (Supplementary Materials).

#### 4.3.2. MIC and Minimal Bactericidal Concentration (MBC) Determination

The antimicrobial activity of Hylin-a1, AR-23, and RV-23 was investigated using a microdilution assay as reported by the National Committee on Clinical Laboratory Standards (NCCLS) guidelines against a broad spectrum of Gram-negative bacteria. Several non-cytotoxic concentrations (from 50 to 0.39 μM) of three peptides were evaluated. In 96-well, the standardized bacterial inoculum (5 × 10^5^ colony forming units, CFU/mL) was inoculated together with AMPs and incubated at 37 °C for 20 h. After the time of incubation, optical density (OD) was measured at TECAN, and data were expressed as a percentage of inhibition. In addition to assessing the MIC values, the bactericidal effect was also studied as reported elsewhere [40].

#### 4.3.3. Time-Kill Kinetics Assay

Using the same conditions as the MIC assay, a time–kill test was performed to monitor bacterial growth over time. Several time intervals (0, 1, 2, 3, 6, and 18 h) were analyzed. Briefly, a serial dilution of each peptide, corresponding to 1/2× MIC, 1× MIC, and 2× MIC, was spotted on Mueller Hinton (MH) agar and incubated at 37 °C overnight (O/N). Finally, bacterial colonies were counted, and the CFU/mL was calculated.

### 4.4. Antibiofilm Activity

#### Attachment, Inhibition, and Degradation Assays

The anti-biofilm activity of peptides was evaluated against *E. coli* and *K. pneumoniae* ATCC strains. Bacterial cultures were grown overnight, then diluted to an OD600 of 0.1 in MH broth supplemented with 1% (*v*/*v*) glucose. A 100 μL aliquot of the bacterial suspension was combined with an equal volume of peptides at non-cytotoxic concentrations in a 96-well plate and incubated at 37 °C for 2 h. Following incubation, non-adherent planktonic cells were removed by washing with phosphate-buffered saline (PBS, Microtech), and adherent cells were incubated with MTT solution for 3 h at 37 °C. Additionally, adherent cells were collected, resuspended in PBS, and plated on agar for viable cell counting. Serial dilutions were performed for each peptide, and CFU/mL were determined after 20 h of incubation at 37 °C (Figure S2) [66]. Absorbance was measured at 570 nm to evaluate adhesion percentage.

To further assess biofilm inhibition and degradation, a colorimetric crystal violet (CV) assay was conducted. Bacterial inocula were prepared and diluted to OD600 values of 0.1 and 0.2 for inhibition and degradation assays, respectively. In the inhibition assay, 100 μL of bacterial suspension was mixed with 100 μL of peptides and incubated for 20 h in a 96-well plate. The wells were then washed with PBS, stained with 0.1% CV for 30 min, and solubilized with ethanol for another 30 min. For biofilm degradation, bacterial inocula were first allowed to form biofilms in a 96-well plate over 20 h, after which 200 μL of peptides were added to each well. Following an additional 20 h incubation at 37 °C, wells were washed with PBS to remove non-adherent cells before staining and solubilization, as described above. Biofilm inhibition and degradation were quantified with spectrophotometric readings at 570 nm, with results expressed as percentages relative to control conditions [67].

### 4.5. Evaluation of Synergistic Activity of AMPs and Meropenem Against E. coli and K. pneumoniae

The checkerboard test was carried out to assess the antibacterial activity of peptides in combination with antibiotics. The test was performed by preparing the bacterial inoculum in the same manner as the tests described above. Non-toxic concentrations of all peptides and serial dilutions of meropenem were inoculated in a 96-well plate either alone or in combination. After 20 h, the plate was read at OD600, and the Fractional Inhibitory Concentration (FICI) was calculated according to the following formula:

FICI = FIC (A) + FIC (B)

FIC (A) = MIC peptide in combination/MIC peptide alone

FIC (B) = MIC meropenem in combination/MIC Meropenem alone

The results have been interpreted according to the values reported in Table 4 [47].

### 4.6. CD Analysis

CD spectra of the peptide at a concentration of 50 μM in 5 mM sodium phosphate buffer (pH 7.4) were recorded in the wavelength range of 260 to 190 nm using a JASCO-705 CD spectrophotometer (Jasco International Co. Ltd., Tokyo, Japan) at a controlled temperature of 25 °C. Each spectrum was collected by averaging 2 scans using a 0.1 cm path length cuvette with a scan speed of 50 nm/min, a response time of 1 s, and a bandwidth of 1 nm. For titration experiments, both the peptides and *E. coli* O128:B12 LPS (Sigma-Aldrich, St. Louis, MO, USA) were solved in 5 mM sodium phosphate buffer at pH 7.4. Peptides maintained at 50 μM were titrated with increasing concentrations of LPS (from 0.2 to 10 μM). Each spectrum was recorded as described above and corrected by subtracting the LPS spectrum at the specific concentration. Graphs were generated using GraphPad Prism 5.1 software (GraphPad Software, San Diego, CA, USA).

### 4.7. Serum Stability Assay

Peptides were mixed with 10% FBS at a final concentration of 0.2 mg/mL and incubated in a water bath at 37 °C. An amount of 50 μL of each sample taken after 0, 30 min, 1 h, 2 h, 4 h, 6 h, and 16 h were mixed with 100 μL of 90% ethanol and incubated on ice for 15 min. Then, the mixture was diluted with 0.1% TFA in H_2_O (1:1). Samples were characterized using LC-MS on a C18 Aeris Peptide column (100 × 2.1 mm), applying a linear gradient from 5 to 80% solvent B (0.1% TFA in CH_3_CN) over solvent A (0.1% TFA in water) in 15 min at a flow rate of 0.2 mL/min. All stability tests were performed at least in triplicate. LC-MS analyses were performed using an Agilent 1260 Infinity II LC/MSD system (Milan, Italy).

### 4.8. Statistical Analysis

All experiments were conducted in triplicate and reported as mean ± standard deviation (SD) calculated with GraphPad Prism (version 8.0.1). Statistical analysis was performed using One-way ANOVA followed by Dunnett’s multiple comparisons test. *p* values  < 0.001 was considered significant.

## 5. Conclusions

In summary, our study focuses on three key innovations: (1) the use of Hylin-a1, AR-23, and RV-23 against MDR strains, including Gram-negative clinical isolates resistant to multiple antibiotics; (2) their effectiveness against bacterial biofilm producers; and (3) the synergistic effect of Hylin-a1, AR-23, and especially RV-23 with meropenem, aiming to reduce antibiotic doses and combat antibiotic resistance. These peptides are very promising peptide agents due to their low cytotoxicity, moderate serum stability, and their ability to interact with the lipid bilayer of the bacterial membrane, destabilizing it and causing its rupture. Future investigations are required to study their efficacy in vivo systems to develop new antibacterial drugs.

## Figures and Tables

**Table 1:** MIC and MBC values of peptides against ATCC bacterial strains. The values were expressed in μM. N/D stands for not detected antibacterial activity in our experimental conditions.

	*K. pneumoniae* ATCC 10031		*E. coli* ATCC 25992	
	MIC (μM)	MBC (μM)	MIC (μM)	MBC (μM)
Hylin-a1	6.25	6.25	25	25
AR-23	6.25	25	25	N/D
RV-23	6.25	12.5	12.5	12.5

**Table 2:** MIC and MBC values of peptides tested against carbapenemase-producer bacteria. The values are expressed in μM. N/D signifies that no antibacterial activity was detected.

Bacterial Strains	AMPs					
	Hylin-a1		AR-23		RV-23	
	MIC (μM)	MBC (μM)	MIC (μM)	MBC (μM)	MIC (μM)	MBC (μM)
*K. pneumoniae* 1711	25	50	50	N/D	12.5	12.5
*K. pneumoniae* 311	N/D	N/D	N/D	N/D	50	50
*K. pneumoniae* 1745	50	50	50	N/D	12.5	25
*K. pneumoniae* 1746	25	N/A	25	N/D	12.5	N/D
*E. coli* 2267	25	50	25	N/D	12.5	12.5
*E. coli* 3140	50	N/D	50	N/D	25	N/D
*E. coli* 716	25	N/D	25	50	25	25
*E. coli* 1376	25	N/D	25	50	6.25	6.25
*E. coli* 1441	50	N/D	25	N/D	6.25	12.5

**Table 3:** *K. pneumoniae*- and *E. coli*-resistant clinical isolates. The resistance patterns and source of isolation are specified for all the isolates used in the present study.

Clinical Isolates	Carbapenemase	Source of Isolation
*K. pneumoniae* 311	OXA-48 (class D)	Rectal swab
*K. pneumoniae* 1746	VIM (class B)	Rectal swab
*K. pneumoniae* 1745	KPC (class A)	Ulcer
*K. pneumoniae* 1711	KPC (class A)	Rectal swab
*E. coli* 2267	NDM (class B)	Rectal swab
*E. coli* 3140	OXA-48 (class D)	Urine
*E. coli* 716	IMP1 (class B)	Urine
	VIM (class B)	
	NDM (class B)	
*E. coli* 1376	NDM (class B)	Rectal swab
*E. coli* 1441	KPC (class A)	Rectal swab

**Table 4:** FICI index values and combinatory effect.

FICI Values	Effect
<0.5	Synergy
0.5 ≤ FIC < 1	Partial synergy
1 ≤ FIC < 4	Additive effect or indifference
4 ≤ FIC	Antagonism

## Data Availability

The original contributions presented in this study are included in the article/Supplementary Materials. Further inquiries can be directed to the corresponding author.

## Supplementary Materials

The following supporting information can be downloaded at: https://www.mdpi.com/article/10.3390/antibiotics14040374/s1, Figure S1: Anti-biofilm activity of AMPs. Figure S2. Viable cell count analysis in biofilm attachment assay. Table S1. Antibiotic resistance profile of *K. pneumoniae* and *E. coli* clinical isolates.
