# Supplementary material for: Frog Skin Peptides Hylin-a1, AR-23, and RV-23: Promising Tools Against Carbapenem-Resistant Escherichia coli and Klebsiella pneumoniae Infections
Source: Antibiotics (Basel). 2025 Apr 3;14(4):374. doi: 10.3390/antibiotics14040374 (PMC12024264; doi:10.3390/antibiotics14040374)
Supplement: Supplementary file 1 [file antibiotics-14-00374-s001.zip › antibiotics-3516141-supplementary.pdf]

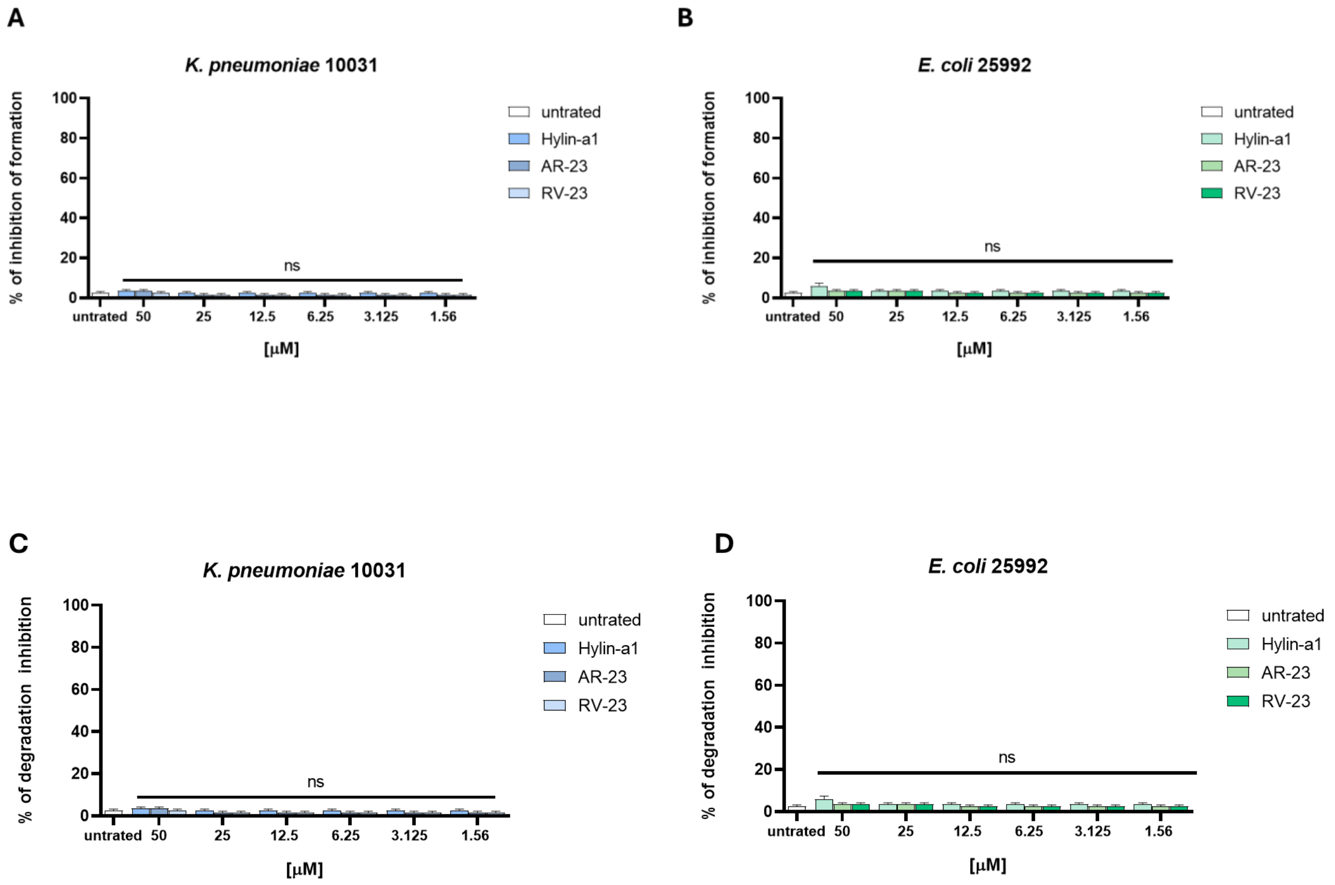

Figure S1. Anti-biofilm activity of AMPs. Biofilm formation of *K. pneumoniae* **(A)** and *E. coli* (**B**); Biofilm degradation of *K. pneumoniae* **(C)** and *E. coli* (**D**). ns: non-significant

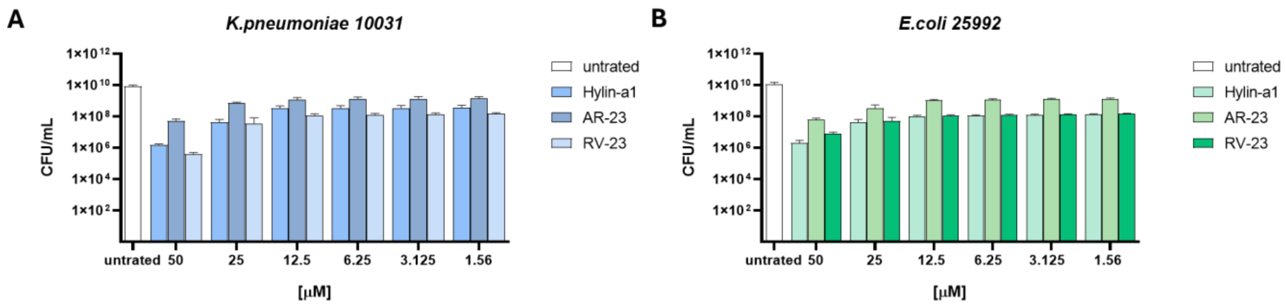

Figure S2. Viable cell count analysis in biofilm attachment assay. Determination of viable cells by counting colony-forming units/mL (CFU/mL). *K. pneumoniae* (A) and *E. coli* (B).

Table S1. Antibiotic resistance profile of *K. pneumoniae* and *E. coli* clinical isolates . **MIC**: Minimum Inhibitory Concentration was expressed in μg/mL; **S**: Sensible; **I**: Intermediate; **R**: Resistant.

**Antibiotic Resistance Profile of the Clinical Isolated Bacteria**		
K. pneumoniae 1711		
**Antibiotics**	**MIC (μg/mL)**	**Interpretation**
Amikacin	≤ 4	S
Amoxicillin/clavulanate	> 32/2	R
Ampicillin	> 8	R
Aztreonam	> 16	R
Cefepime	> 8	R
Ceftazidime	> 16	R
Ceftriaxone	> 4	R
Cefuroxime	> 8	R
Ciprofloxacin	> 1	R
Ertapenem	> 1	R
Fosfomycin	32	S
Gentamicin	> 4	R
Imipenem	> 8	R
Levofloxacin	> 1	R
Meropenem	> 16	R
Piperacillin/tazobactam	> 64/4	R
Temocillin	> 32	R
Tobramycin	> 4	R
Trimethoprim/sulfamethoxazole	> 4/76	R
Ceftazidime-avibactam	> 8/4	R
Ceftolozano tazobactam	> 4/4	R
Cefiderocol		I
Meropenem-vaborbactam	> 8/8	R
K. pneumoniae 311		
**Antibiotics**	**MIC (μg/mL)**	**Interpretation**
Amikacin	≤ 4	**S**
Amoxicillin/clavulanate	> 32/2	R
Ampicillin	> 8	R
Aztreonam	> 16	R
Cefepime	> 8	R
Ceftazidime	> 16	R
Ceftriaxone	> 4	R
Cefuroxime	> 8	R

Ciprofloxacin	> 1	R
Ertapenem	> 1	R
Fosfomycin	≤ 16	S
Gentamicin	> 4	R
Imipenem	2	S
Levofloxacin	> 1	R
Piperacillin/tazobactam	> 64/4	R
Temocillin	> 32	R
Tobramycin	> 4	R
Trimethoprim/sulfamethoxazole	2/38	S
Ceftazidime-avibactam	1/ 4	S
Ceftolozano tazobactam	> 4/4	R
Meropenem-vaborbactam	4/ 8	S
K. pneumoniae 1745		
**Antibiotics**	**MIC (μg/mL)**	**Interpretation**
Amikacin	8	S
Amoxicillin/clavulanate	> 32/2	R
Ampicillin	> 8	R
Aztreonam	> 16	R
Cefepime	> 8	R
Ceftazidime	> 16	R
Ceftriaxone	> 4	R
Cefuroxime	> 8	R
Ciprofloxacin	> 1	R
Ertapenem	> 1	R
Fosfomycin	≤ 16	S
Gentamicin	> 4	R
Imipenem	> 8	R
Levofloxacin	> 1	R
Meropenem	> 16	R
Piperacillin/tazobactam	> 64/4	R
Temocillin	> 32	R
Tobramycin	> 4	R
Trimethoprim/sulfamethoxazole	> 4/76	R
Ceftazidime-avibactam	> 8/4	R
Ceftolozano tazobactam	> 4/4	R
Meropenem-vaborbactam	> 8/8	R
K. pneumoniae 1746		
**Antibiotics**	**MIC (μg/mL)**	**Interpretation**
Amikacin	≤ 4	S
Amoxicillin/clavulanate	> 32/2	R
Ampicillin	> 8	R
Aztreonam	≤ 1	S
Cefepime	8	R
Ceftazidime	> 16	R
Ceftriaxone	> 4	R
Ciprofloxacin	0.5	I
Colistin	0.25	S
Ertapenem	> 1	R
Fosfomycin	≤ 16	S
Gentamicin	2	S
Imipenem	4	I
Levofloxacin	1	I
Meropenem	1	S
Piperacillin/tazobactam	32/4	R
Tobramycin	> 4	R
Trimethoprim/sulfamethoxazole	≤ 1/19	S
Ceftazidime-avibactam	> 8/4	R
Ceftolozano tazobactam	> 4/4	R
Cefiderocol		S
Meropenem-vaborbactam	≤ 2/8	S
E. coli 2267		

Antibiotics	MIC (µg/mL)	Interpretation
Amikacin	≤ 4	S
Amoxicillin/clavulanate	> 32/2	R
Ampicillin	> 8	R
Aztreonam	> 16	R
Cefepime	8	R
Ceftazidime	> 16	R
Ceftriaxone	> 4	R
Cefuroxime	> 8	R
Ciprofloxacin	> 1	R
Ertapenem	> 1	R
Fosfomycin	≤ 16	S
Gentamicin	≤ 1	S
Imipenem	> 8	R
Levofloxacin	> 1	R
Meropenem	> 16	R
Piperacillin/tazobactam	> 64/4	R
Temocillin	> 32	R
Tigecycline	> 2	R
Tobramycin	≤ 1	S
Trimethoprim/sulfamethoxazole	> 4/76	R
Ceftazidime-avibactam	8/4	S
Ceftolozano tazobactam	> 4/4	R
Meropenem-vaborbactam	> 32/2	R
E. coli 3140		
**Antibiotics**	**MIC (µg/mL)**	**Interpretation**
Amikacin	≤ 4	S
Amoxicillin/clavulanate	> 32/2	R
Ampicillin	> 8	R
Aztreonam	≤ 1	S
Cefepime	≤ 1	S
Ceftazidime	16	R
Ceftriaxone	≤ 0.5	S
Cefuroxime	> 8	R
Ciprofloxacin	≤ 0.125	S
Ertapenem	> 1	R
Gentamicin	≤ 1	S
Imipenem	2	S
Levofloxacin	≤ 0.25	S
Meropenem	0.5	S
Nitrofurantoin	≤ 16	S
Norfloxacin	≤ 0.5	S
Piperacillin/tazobactam	> 64/4	R
Temocillin	> 32	R
Tobramycin	≤ 1	S
Trimethoprim/sulfamethoxazole	≤ 1/19	S
Ceftazidime-avibactam	≤ 0.25/4	S
Ceftolozano tazobactam	> 4/4	R
Meropenem-vaborbactam	≤ 2/8	S
E. coli 716		
**Antibiotics**	**MIC (µg/mL)**	**Interpretation**
Amikacin	≤ 4	S
Amoxicillin/clavulanate	> 32/2	R
Ampicillin	> 8	R
Aztreonam	> 16	R
Cefepime	8	R
Ceftazidime	> 16	R
Ceftriaxone	> 4	R
Cefuroxime	> 8	R
Ciprofloxacin	> 1	R
Ertapenem	> 1	R
Fosfomycin	≤ 16	S

Gentamicin	≤ 1	S
Imipenem	> 8	R
Levofloxacin	> 1	R
Meropenem	> 16	R
Piperacillin/tazobactam	> 64/4	R
Temocillin	> 32	R
Tigecycline	> 2	R
Tobramycin	≤ 1	S
Trimethoprim/sulfamethoxazole	> 4/76	R
Ceftazidime-avibactam	8/4	S
Ceftolozano tazobactam	> 4/4	R
Meropenem-vaborbactam	> 32/2	R
	E. coli 1376	
**Antibiotics**	**MIC (µg/mL)**	**Interpretation**
Amikacin	> 16	R
Amoxicillin/clavulanate	> 32/2	R
Ampicillin	> 8	R
Aztreonam	> 16	R
Cefepime	> 8	R
Ceftazidime	> 16	R
Ceftriaxone	> 4	R
Cefuroxime	> 8	R
Ciprofloxacin	> 1	R
Ertapenem	> 1	R
Fosfomycin	> 64	R
Gentamicin	> 4	R
Imipenem	> 8	R
Levofloxacin	> 1	R
Meropenem	> 16	R
Piperacillin/tazobactam	> 64/4	R
Temocillin	> 32	R
Tigecycline	≤ 0.5	S
Tobramycin	> 4	R
Trimethoprim/sulfamethoxazole	> 4/76	R
Ceftazidime-avibactam	≤ 0.25/4	S
Ceftolozano tazobactam	> 4/4	R
Meropenem-vaborbactam	> 8/8	R
	E. coli 1441	
**Antibiotics**	**MIC (µg/mL)**	**Interpretation**
Amikacin	≤ 4	**S**
Amoxicillin/clavulanate	> 32/2	R
Ampicillin	> 8	R
Aztreonam	> 16	R
Cefepime	8	R
Ceftazidime	> 16	R
Ceftriaxone	> 4	R
Cefuroxime	> 8	R
Ciprofloxacin	≤ 0.125	S
Ertapenem	> 1	R
Fosfomycin	≤ 16	S
Gentamicin	≤ 1	S
Imipenem	4	I
Levofloxacin	≤ 0.25	S
Meropenem	1	S
Piperacillin/tazobactam	> 64/4	R
Temocillin	8	S
Tigecycline	≤ 0.5	S
Tobramycin	≤ 1	S
Trimethoprim/sulfamethoxazole	≤ 1/19	S
Ceftazidime-avibactam	≤ 0.25/4	S
Ceftolozano tazobactam	> 4/4	R
Meropenem-vaborbactam	≤ 2/8	S

antibiotics
